# Activation of EP4 receptor limits transition of acute to chronic heart failure in lipoxygenase deficient mice

**DOI:** 10.7150/thno.51183

**Published:** 2021-01-01

**Authors:** Vasundhara Kain, Kevin A. Ingle, Namakkal S. Rajasekaran, Ganesh V. Halade

**Affiliations:** 1 Division of Cardiovascular Sciences, Department of Medicine, University of South Florida.; 2 Division of Nephrology, The University of Alabama at Birmingham, Alabama.; 3 Department of Pathology, The University of Alabama at Birmingham, Alabama.

**Keywords:** Inflammation, macrophages, leukocytes, resolution, cardiac remodeling, heart failure

## Abstract

**Aim:** Immune responsive 12/15 lipoxygenase (12/15LOX)-orchestrate biosynthesis of essential inflammation-resolution mediators during acute inflammatory response in post-myocardial infarction (MI). Lack of 12/15LOX dampens proinflammatory mediator 12-(S)-hydroxyeicosatetraenoic acid (12-(S)-HETE), improves post-MI survival, through the biosynthesis of endogenous mediators epoxyeicosatrienoic acids (EETs; cypoxins) to resolve post-MI inflammation. However, the mechanism that amplifies cypoxins-directed cardiac repair in acute heart failure (AHF) and chronic HF (CHF) remains of interest in MI-directed renal inflammation. Therefore, we determined the role of EETs in macrophage-specific receptor activation in facilitating cardiac repair in 12/15LOX deficient mice experiencing HF.

**Methods and Results:** Risk-free young adult (8 -12 week-old) male C57BL/6J wild-type mice (WT; n = 43) and 12/15LOX^-/-^ mice (n = 31) were subjected to permanent coronary artery ligation and monitored at day (d)1, d5 (as acute HF), and d28 to d56 (8 weeks; chronic HF) post-surgery maintaining no-MI mice that served as d0 naïve controls. Left ventricle (LV) infarcted area of 12/15LOX^-/-^ mice displayed an increase in expression of prostanoid receptor EP4 along with monocyte chemoattractant protein-1 CCL2 in AHF and CHF. The transcriptome analysis of isolated leukocytes (macrophages/neutrophils) from infarcted LV revealed a higher expression of EP4 on reparative macrophages expressing *MRC-1* in 12/15LOX^-/-^ mice. Deletion of 12/15LOX differentially modulated the miRNA levels, downregulating miR-23a-3p (~20 fold; *p* < 0.05) and upregulating miR-125a-5p (~160 fold; *p* < 0.05) in AHF which promoted polarization of the macrophages towards reparative phenotype. Furthermore, 12/15LOX deletion markedly attenuated renal inflammation with reduced levels of NGAL and KIM-1 and apoptotic markers in the kidney during CHF.

**Conclusion:** In risk-free mice during physiological cardiac repair, absence of 12/15LOX promoted reparative macrophages with marked activation of EP4 signaling thereby improving post-MI survival and limiting renal inflammation in acute and advanced HF. The future studies are warranted to advance the role of EETs in macrophage receptor biology.

## Introduction

Heart failure (HF) is a life-threatening end stage disease that claims more than 26 million lives worldwide 1. The American College of Cardiology (ACC)/American Heart Association (AHA) classified HF into A, B, C, and D stages, sequenced from mild to severe symptoms of HF at rest or upon minimal physical exertion 2. Globally, 7-45% of patients with HF die within 1 year of hospitalization and >50% die within 5 years 1. Myocardial infarction (MI) triggers an intense inflammatory response with an overlapping resolving response. If an acute inflammatory phase overrides the resolving phase, it can lead to chronic inflammation that progresses towards the HF 3-5. Thus, to develop treatment strategy and to identify appropriate target, it is necessary to focus on both acute and chronic HF. For simplicity, ischemia-induced progressive HF can be categorized into two forms: 1) AHF (Acute Heart Failure) and 2) CHF (Chronic Heart Failure). The development of acute HF is rapid due to the severe damage to the functional myocardium after ischemic insult and an intense leukocyte influx. Progressive HF is a major clinical complication that encompasses pathological remodeling of the heart with the possible necessity of heart transplant.

After ischemia, leukocyte directed innate immune response is dynamic and unsteady that create a bimodal response with an initial wave called as an early phase or acute inflammatory response which occurs within 24 - 72 h 6. The second wave is called a reparative wave, which compensates for time-dependent dilatation, the distortion of ventricular shape, and mural hypertrophy 7. However, the second wave of the bimodal response, if not repaired or controlled in a timely manner, leads to the progression of CHF. Since, 12/15lipoxygenase (12/15LOX)-derived pro-inflammatory mediators amplify the acute inflammatory response after MI, 8 our previous results indicate that 12/15LOX deficiency promotes effective resolution in acute HF 9, 10. However, the role of 12/15LOX in CHF with renal inflammation is undetermined. A recent study has shown that the deletion of 12/15LOX improves survival with the increase in CYP2J-derived epoxyeicosatrienoic acids (EETs) in HF survivor mice 9. Given that EETs are increased in the spleen and infarcted heart, thus we uncovered the possible molecular mechanism that how EETs have a positive impact on cardiac function 11, 12. In the current study, we determined whether prostanoid receptors EP4/EP2 activation plays protective role in receptor-cell specificity during the acute and chronic stages of HF post-MI. Our results suggest that deletion of the 12/15LOX activates EP4 receptor on reparative macrophages and attenuates renal inflammation, thereby improves function, and delays progression of CHF post-MI.

## Methods

### Animal care compliance

All animal procedures were conducted according to the “Guide for the Care and Use of Laboratory Animals” (8^th^ Edition. 2011), AVMA Guidelines for the Euthanasia of Animals: (2013 Edition) and were approved by the Institutional Animal Care and Use Committees at the University of South Florida, Florida and the University of Alabama at Birmingham, Alabama, USA.

### Mice and coronary ligation surgery

Risk free 8-12 weeks C57BL/6 (wild type; WT; Cat # 000664 ) and 12/15 LOX null mice (12/15LOX^-/-^(B6.129S2-Alox15^tm1Fun^/J; Cat # 002778) on the C57BL/6 genetic background were obtained from the Jackson Laboratory (Bar Harbor, Maine, USA) and were maintained under constant temperature (19.8 - 22.2 °C). The mice were given free access to water and standard chow diet. Both WT and 12/15LOX^-/-^ were subjected to the surgical coronary ligation of the left anterior descending coronary artery, as described previously and monitored everyday two time till designed CHF end stage (day 56) 13, 14. In brief, the mice were anesthetized with 2-4% isoflurane, and the left anterior descending coronary artery was permanently ligated using minimally invasive surgery. Before MI surgery, carprofen (5 mg/kg SQ) and buprenorphine (0.1 mg/kg SQ) were administered to reduce pain and after 24 hours (h) of surgery signs of AHF confirmed using high resolution echocardiography 15.

### Autopsy and post-MI survival analysis

The mice were checked daily (two-time morning and evening) for 56 days for post-MI survival analysis. The presented survival curve is a cumulative curve from previous study to establish that the survival of 12/15LOX^-/-^ mice in acute and chronic HF 9, 10. Mice which died within 24 h after ligation surgery are considered as perioperative mortality and excluded from the survival curve. The cardiac rupture was confirmed if a large blood slit in the thoracic cavity and the LV rupture site were observed 16. To confirm that all mice developed CHF, echocardiography was performed and exclusion-inclusion criteria are detailed in echocardiography procedure.

### High resolution echocardiography

For the echocardiography analysis, mice were anesthetized using 0.8-1.0% isoflurane in a 100% oxygen mix. Electrocardiograms and heart rates were monitored using a surface electrocardiogram. Images were acquired using the Vevo 2100 and 3100 in the vivo imaging system (Visual Sonics) equipped with probes up to 40 MHz and a resolution of 30 µm. Short and long axis images were acquired at heart rates > 400 beats/min to achieve physiologically relevant measurements. Measurements were taken from the two-dimensional parasternal long-axis (B-mode) and short-axis (m mode) recordings from the mid-papillary region. Echocardiographic studies were performed before necropsy for the day (d) 0 control mice and d1, d5, d28, and d56 post-MI mice. For each variable, three images from consecutive cardiac cycles were measured and averaged by an operator blinded to genotype 9. Mice that showed fractional shortening more than 10% at d1 post-MI were excluded from survival curve indicative of inaccurate or misplaced ligation.

### Necropsy and infarct area analysis

Tissue and blood samples were collected as previously described 9. The infarct area was calculated as the percentage of infarct area to total LV area 17.

### Isolation of neutrophils (NΦ), and macrophages (MΦ) from LV infarct

To distinguish resolve and non-resolving phenotypes of NΦ and MΦ were isolated from LV infarct at d1 and d5 post-MI using Ly6G^+^ and CD11b^+^ -specific magnetic beads using the detailed protocol described previously 17.

### Isolation of peritoneal MΦs and milieu dependent differentiation *in vitro*

To determine molecular mechanism, peritoneal MΦ were isolated from mice peritoneum as described previously 18. Next, MΦ were plated in a 12‐well plate (1 × 10^6^ cells/well) on coverslips, incubated at 37 °C overnight to allow the cells to adhere, and subsequently washed with fresh media to remove any unattached cells. Then plated cells were treated with LPS (1 µg/ml) + IFN-g (20 ng/ml) and IL-4 (20 ng/mL) +IL-13 (20 ng/mL) for 4 h to differentiate them as MΦ1 and MΦ2 respectively. EP4 agonist-CAY10598 (100 nM) was used as positive control. MΦ were treated with CAY10598 for 2 h. Cells (peritoneal MΦ) were fixed using 4% paraformaldehyde, permeabilized using 0.1% triton and blocked for 1 h in 10% goat serum. Peritoneal MΦ were subsequently incubated with EP4‐antibody (Santa Cruz biotechnology) overnight and probed with an Alexa‐555 secondary antibody (Molecular Probe), each for 60 min at room temperature. The nucleus was stained using Hoechst (Molecular Probe). Cells were mounted using anti‐fade mounting media (ThermoFisher Scientific) and then visualized and photographed using Nikon A1 high‐speed laser confocal microscope.

### Isolation of fibroblast and myofibroblast differentiation

To determine cell-specific receptor, cardiac fibroblasts (CF) were isolated from the LV, and myo-fibroblast differentiation was induced using TGF-β as described previously 18 and stained for EP4 expression as described in above protocol.

### Histology, and confocal microscopy

The confocal microscopy was performed on LV mid-cavity, kidney frozen section, MΦ and CF using EP4 antibody (Abcam) as previously described 19. To measure the area of cardiomyocyte, Alexa Fluor^®^ 488 conjugate of WGA (wheat gram agglutinin) was used as previously described 20.

### Collagen staining using picrosirius red staining

For collagen deposition measurement particularly in the infarcted area, the LV stained using picrosirius red (PSR) staining as detailed in previous report 20.

### Image analysis for IHC, PAS, and PSR staining

The image analysis was done with a microscope (BX43) using the cellSens Dimension program (Olympus 1.9), then analyzed for percent area stained using Image-Pro Premier 64-bit software 20.

### LV and kidney protein extraction and immunoblotting

Protein extraction and immunoblotting for EP4, EP2, and CCL2 on LV and NGAL for kidney tissues from d0 to d56 was performed as described previously 16. The total protein lane was used as an internal loading control. Image J was used for densitometry analysis.

### Quantitative Real-Time PCR

For qPCR, reverse transcription was performed with 2.0 μg of total RNA using the SuperScript^®^ VILO cDNA Synthesis Kit (Invitrogen, CA, USA). Quantitative PCR *for EP4, EP2, CCL2, MRC-1, TNF-α, TREM,* and *neutrophil gelatinase-associated lipocalin (NGAL)*, genes were performed using TaqMan probes (Applied Biosystems, CA, USA) on master cycler ABI, 7900HT. Gene levels were normalized to Hprt-1 as the housekeeping control gene. The results were reported as 2^-ΔCt^ (ΔΔCt) values. All the experiments were performed in duplicates with n = 5 / group.

### Infarcted heart and naïve control miRNA array profiling

Micro (mi) RNA gene (miRNA) gene expression profiling was performed using miR PCR arrays in frozen infarcted LV tissue as per the manufacturer's instructions and previous reports 21. In brief, miRs was isolated with the miRNeasy Mini Kit (217004; Qiagen, Germantown, MD, USA), and cDNA synthesis was performed with miScript II RT Kit (Qiagen). Each sample was loaded on the RT^2^-PCR plate (mi-Script miRNA PCR array MIMM-113ZE-4; Qiagen) and was run on an ABI 7900HT PCR system (Thermo Fisher Scientific). The results were reported as 2^-ΔΔCt^ values.

### Plasma creatinine

Plasma creatinine levels for no-MI day 0 naïve controls and days 1, 5, 28, and 56 post-MI from WT and 12/15LOX^-/-^ mice were determined using LC-MS/MS as previously described 21.

### Statistical analysis

Data are expressed as mean ± SEM and presented as box-whisker plots (minimum to maximum). Minimum 4 mice/group is used for all the experiments except immunoblots where 2-3 mice/group is used. Statistical analyses were performed using Graphpad Prism 8. Analysis of variance (ANOVA), followed by Newman-Keuls post-hoc test, was used for multiple comparisons. The Kaplan-Meier test and the log-rank test for survival analysis. All immunoblotting densitometry data was normalized to total protein. For 2 group comparison, student t-test (unpaired) was applied and *p* < 0.05 was considered as statistically significant.

## Results

### Abrogation of 12/15LOX delays progression of HF with improved ventricular function in mice

**Figure 1:**
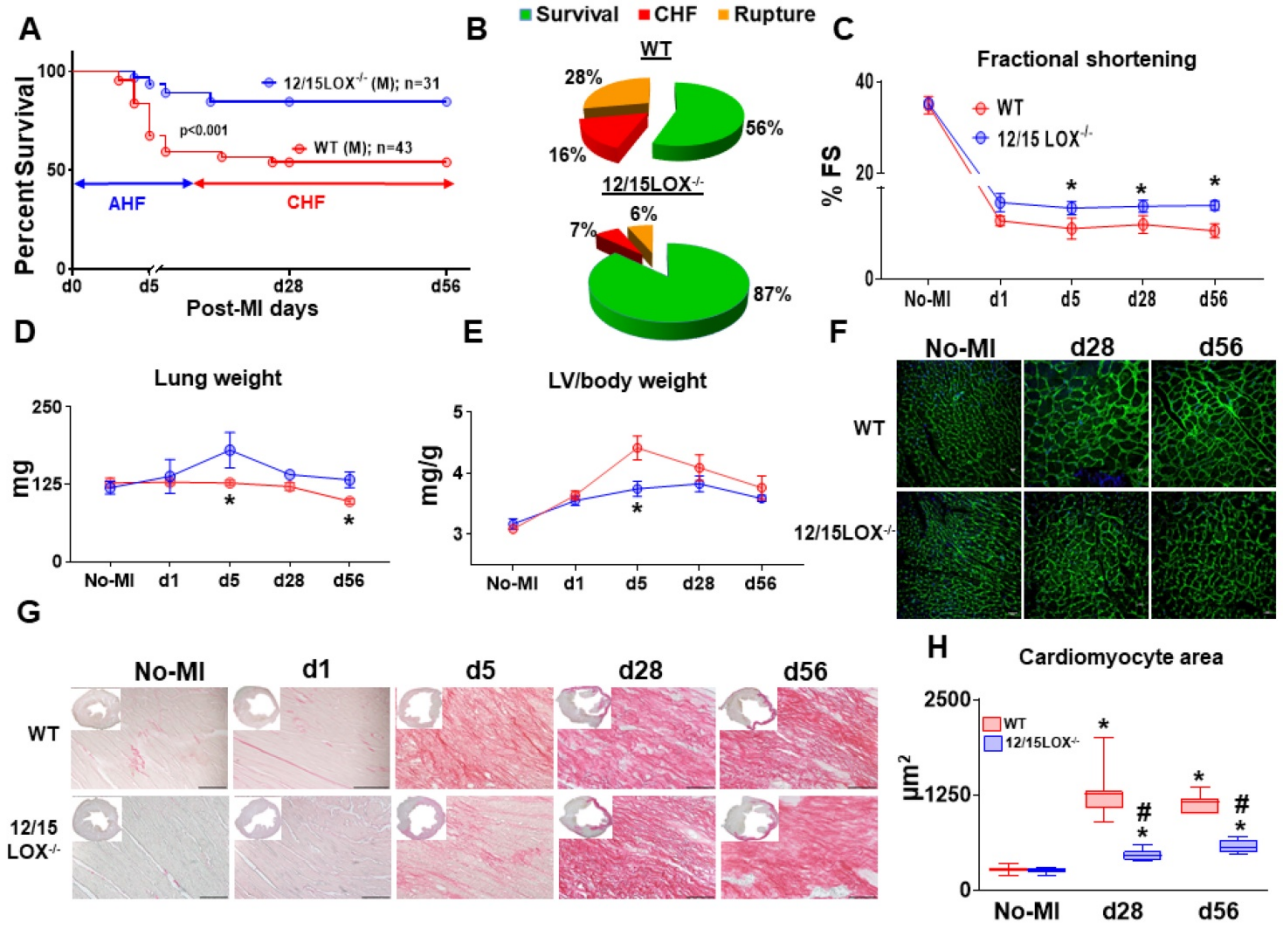
** 12/15LOX^-/-^ mice have improved survival in progression of acute (AHF) to chronic heart failure (CHF) post-MI.** (A) Kaplan-Meier survival curve compared by log-lank test representing improved survival in 12/15LOX^-/-^ male mice post-MI measured in progressive heart failure (d56). *p < 0.001 vs. WT, n = 43 (male) WT and n = 31(male) 12/15LOX^-/-^. (B) Pie chats representing % of survival (green), chronic heart failure (CHF-red) and ruptures (orange) in WT and 12/15 LOX^-/-^ mice at post-MI d56. (C) Temporal data of fractional shortening (D) Lung weight in mg (E) Left ventricle (LV) / body weight ratio. (F) Wheat germ agglutinin (WGA) displaying cardiomyocyte area in LV. (G) LV histology (Picro Sirius Red) images displaying post-MI changes from no-MI to post-MI d56 in WT and 12/15 LOX^-/-^ mice (H) Whisker and box graph representing cardiomyocyte area in no-MI control, post-MI d28, and d56. Values are represented as mean ± SEM. Whisker and box plots showing median and interquartile range. Minimum and maximum values are represented in each group; n = 10/group/day; *p < 0.05 vs d0 respective control, #*p* < 0.05 vs WT at respective time point. Analysis of variance (ANOVA), followed by Newman-Keuls post-hoc test, was used for multiple comparisons.

To validate the post-MI survival as previously reported, first we determined whether 12/15LOX deletion have same efficacy and potency to resolve inflammation in progressive CHF. Previous report showed that deletion of 12/15 LOX improved LV function with effective resolution in AHF 4. To determine the role of 12/15LOX in CHF, we monitored post-MI survival from the AHF (post-MI d1) to CHF (post-MI d56) with an assessment of longitudinal ventricular function. Compared to the WT, 12/15LOX^-/-^ mice displayed higher post-MI survival in CHF (56% in WT vs 87% in 12/15LOX^-/-^ mice; *p*<0.001), with lower rupture rates in CHF **(Figure 1A-B)**. Longitudinal heart function data acquired using high-resolution echocardiography from the HF survivor show improved fractional shortening in 12/15LOX^-/-^ mice compared to WT mice in CHF **(Figure 1C and Table 1)**. The gravimetric analysis **(Figure 1D-E and Table 1)** suggests that LV mass-to-body weight ratio is reduced in 12/15LOX^-/-^ mice with a decreased in lung mass compared with WT mice in CHF. The analysis of cardiomyocyte area using WGA revealed decreased hypertrophy in 12/15LOX^-/-^ mice when compared with WT **(Figure 1F and H)** consistent with a decrease in LV mass-to-body weight ratio in CHF. Histological analysis of LV indicated LV dilation and mature scar formation in CHF, the tissue was stained using H and E (LV mid-cavity; insert) and PSR. Compared with WT mice, 12/15LOX^-/-^ mice showed decrease in collagen levels during acute phase (post-MI d5) indicative of accelerated resolution. In CHF (post-MI d56), qualitative analyses of the collagen density), showed no difference in the PSR stained area in WT and 12/15LOX^-/-^ mice which indicated successful ligation with equal injury during ischemic procedure **(Figure 1G and Figure S1)**. Thus, presented results extends our previous findings in AHF and validates that 12/15LOX deletion improves heart function and survival in CHF with reduced LV remodeling.

### Activation of EP4 receptor directs cardiac repair in 12/15LOX null mice post-MI

**Figure 2:**
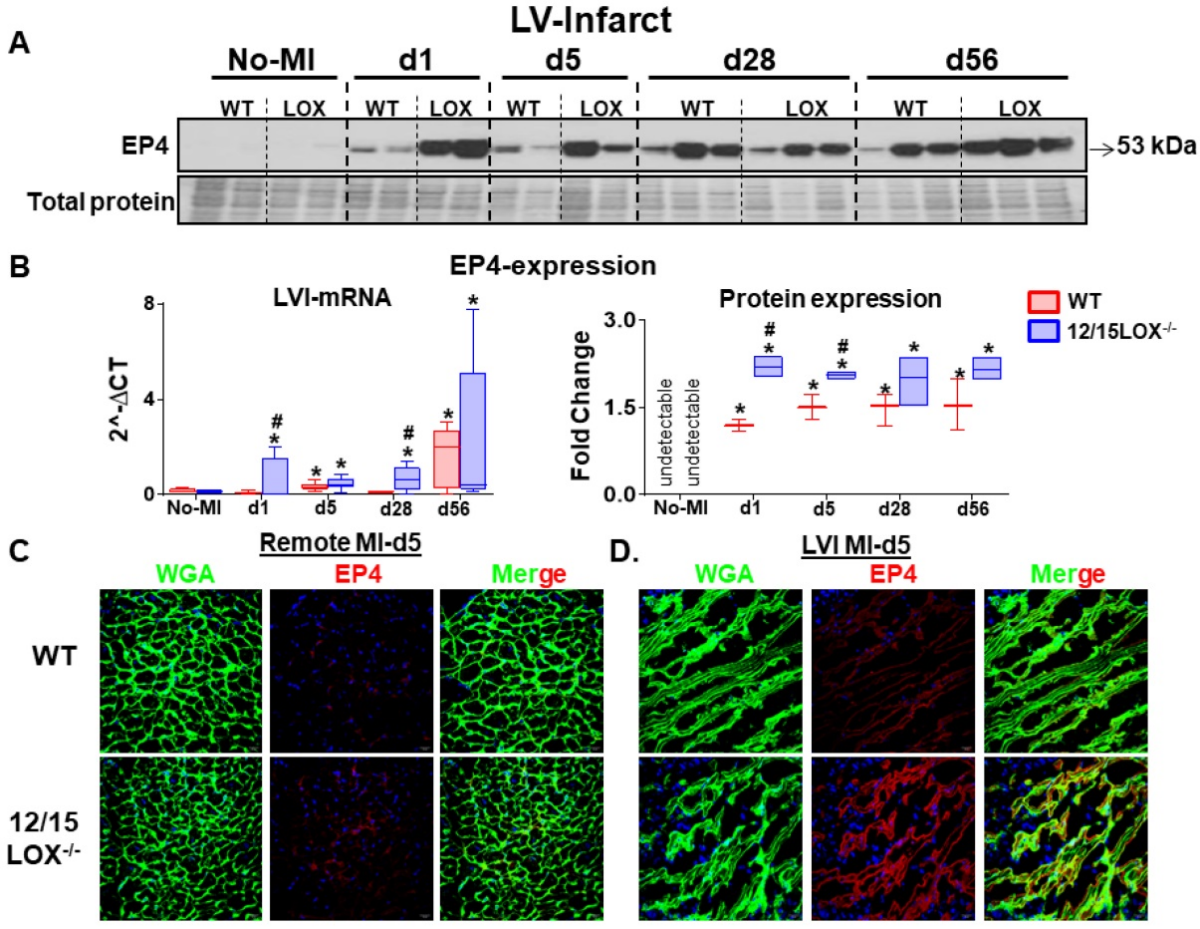
** Activated EP4 directs cardiac repair in 12/15LOX^-/-^ in progressive heart failure post-MI.** (A) Immunoblot showing robust increase in EP4 expression in WT and 12/15LOX^-/-^ mice in in temporal kinetics of HF. (B) mRNA expression and densitometric analysis of EP4 in LV infarct of no-MI and post-MI d1 till d56 in WT and 12/15LOX^-/-^ mice. Values are represented as whisker and box plots show median and interquartile range. Minimum and maximum values are represented in each group by the whiskers of the plot; n = 2 - 5/group/day; *p < 0.05 vs d0 respective control, #*p* < 0.05 vs WT at respective time point. Analysis of variance (ANOVA), followed by Newman-Keuls post-hoc test, was used for multiple comparisons. (C, D) Representative remote and infarct immunofluorescence images displaying cardiomyocyte area by WGA staining (green) and EP4 expression (red) in LV infarct and LV remote of WT and 12/15LOX^-/-^ mice at post-MI d5 (magnification 40×, scale = 20 µm). Images are representative of 5-8 field/slide; n = 4 slide/group.

Activation of prostanoid receptor gives feed forward loop to LOX and cyclooxygenase (COX) metabolites that are endogenously biosynthesized in response to immune response post-ischemia 22. To determine how prostanoid receptor are governed the post-MI ischemia milieu, we examined expression level of the E-type prostanoid receptors EP2 and EP4 in the LV (infarct and remote) under acute and CHF post-MI settings. Both EP2 and EP4 levels were amplified in AHF (post-MI d1) in the infarcted myocardium. Post-ischemia, the EP2 expression peaked at d5 post-MI and then decreased in the CHF at d28 and d56 post-MI. However, no difference was observed in the expression level of EP2 between WT and 12/15LOX^-/-^ mice **(Figure S2A-C)**. EP4 expression, particularly upregulated in acute cardiac repair (d1 and d5; 2.2 - fold, p<0.01) in LV infarct in 12/15LOX^-/-^ mice compared with WT mice of respective MI-controls. In CHF (d28 and d56), no difference was observed in the EP4 expression in the infarcted myocardium of 12/15LOX^-/-^ mice compared with WT mice **(Figure 2A-B)**. Activation of EP4 was further validated by using the immunofluorescence in LV during acute-MI which suggests EP4 expression was significantly increased in the infarct zone, but not in the remote zone **(Figure 2C)**. Thus, our results indicated that EP4 receptor expression is confined to the area of ischemic injury in the process of cardiac repair during early myocardium healing post-MI.

### Expression of EP4 in reparative MΦs facilitates cardiac healing response in the infarcted myocardium

**Figure 3:**
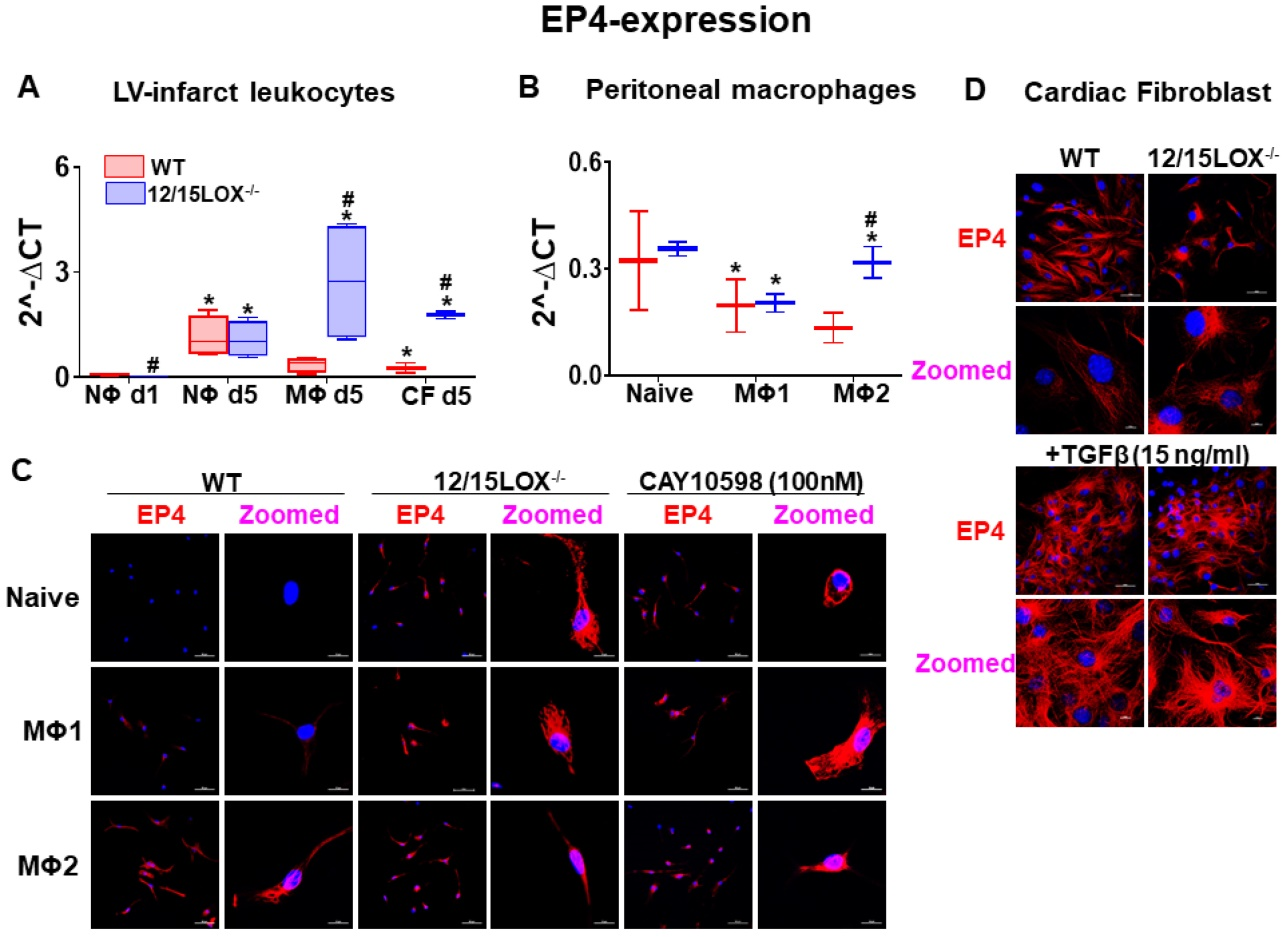
** EP4 expressing MΦs facilitates resolving phenotype in cardiac repair post-MI. (A)** mRNA expression of EP4 in isolated leukocytes at post-MI d1, d5, and in cardiac fibroblast (CF) at post-MI d5 isolated from LV infarct. Values are represented as mean ± SEM; n = 4; *p < 0.05 vs WT at neutrophils at d1, #*p* < 0.05 vs WT at respective time point. **(B)** mRNA expression of EP4 on peritoneal MΦs (Naïve, M1 and M2 phenotype) isolated from WT and 12/15LOX^-/-^ mice. Values are represented as box plots showing median and interquartile range. Minimum and maximum values are represented in each group by the whiskers of the plot; n = 4; *p < 0.05 vs (C) Representative immunofluorescence images displaying EP4 expression (red) and nuclei(blue) on peritoneal MΦ (Naïve, M1 and M2 phenotype) isolated from WT and 12/15LOX^-/-^ mice. WT peritoneal MΦ (naïve, M1 and M2 phenotype) was treated with EP4 agonist (CAY10598 -100 nM) to confirm EP4 stimulation (magnification 40X, scale = 50 µm; zoom = 5.72; scale =10 µm). Images are representative of 5-8 field/slide; n = 4 slide/group. (D) Representative immunofluorescence images displaying EP4 expression (red) and nuclei (blue) on CF and myofibroblast (treated with TGF-β-15 ng/ml for 18 h) isolated from WT and 12/15LOX^-/-^ mice (magnification 40X, scale = 50 µm; zoom = 3.53; scale =10 µm). Images are representative of 5-8 field/slide; n = 4 slide/group. Analysis of variance (ANOVA), followed by Newman-Keuls post-hoc test, was used for multiple comparisons.

During the acute phase of MI, activated leukocytes play a decisive role in cardiac repair of infarcted myocardium 4, 5. Therefore, we isolated leukocytes from infarcted myocardium during the early acute stage i.e. d1 and d5 post-MI and segregated neutrophils (NΦ) and macrophages (MΦ) using magnetic beads. To dissect the possible pattern of EP4/EP2 expression on activated fibroblast, we simultaneously isolated cardiac fibroblasts (CF) along with leukocytes from infarcted LV. Of note, no difference was observed in the expression of EP4 on NΦ isolated from WT and 12/15LOX^-/-^ mice at d1 and d5 post-MI but NΦs have increased level of EP2 at MI-d5 in 12/15LOX^-/-^ mice. Isolated MΦs from 12/15LOX^-/-^ mice revealed higher expression of EP4 and EP2 at d5 post-MI compared with WT. In the reparative phase, CF of 12/15LOX^-/-^ mice expressed higher EP4 and EP2 levels compared with WT-CF at d5 post-MI **(Figure 3A and Figure S2D)**. Since, the MΦs has higher expression of EP4 compared with CF fibroblast therefore, we determined the MΦ phenotype due to EP4 activation. For that, we isolated peritoneal MΦ and differentiated them to classical inflammatory MΦ (M1) and reparative MΦ (M2) *in vitro*. The quantitative RT-PCR suggest enhanced EP4 expression on reparative MΦ compared with classical inflammatory MΦ **(Figure 3B)**, however EP2 was equally expressed on both reparative MΦ and classical inflammatory MΦ **(Figure S2E)**. EP4 protein expression on MΦs was validated using immunofluorescence. The immunofluorescence data indicated that reparative MΦs had higher EP4 expression compared with classical MΦ isolated from WT. Of note, the MΦ isolated from 12/15LOX^-/-^ mice displayed EP4 expression without any stimulus suggestive of inherited reparative capacity due to 12/15LOX deficiency. Further, MΦ expressed EP4 intensely amplified with the classical and reparative stimulus in 12/15LOX^-/-^ mice compared with the WT. To evaluate EP4 specificity, EP4 agonist, CAY10598 (100 nM) was used as a positive control to validate that EP4 is pre-activated in 12/15LOX^-/-^ mice **(Figure 3C)**. To dissect cell-type specificity, EP4 expression was measured in CF after TGF-β activation and differentiation into myofibroblast isolated from WT and 12/15LOX^-/-^ mice. The immunofluorescence data depicted that there is no difference in EP4 expression on CF isolated from WT and 12/15LOX^-/-^ **(Figure 3D)**. Thus, *in vivo* and *in vitro* approaches and comprehensive measurement of EP4 expression on differential cell type of leukocyte and CF indicated reparative role of EP4 is directly linked to the reparative MΦ phenotype in cardiac repair, indendenent of EP4 expression on CF in post-MI.

### Differential expression patterns of miRNAs that regulate cytokines play a crucial role in the transition of acute to chronic HF in 12/15LOX null mice

**Figure 4:**
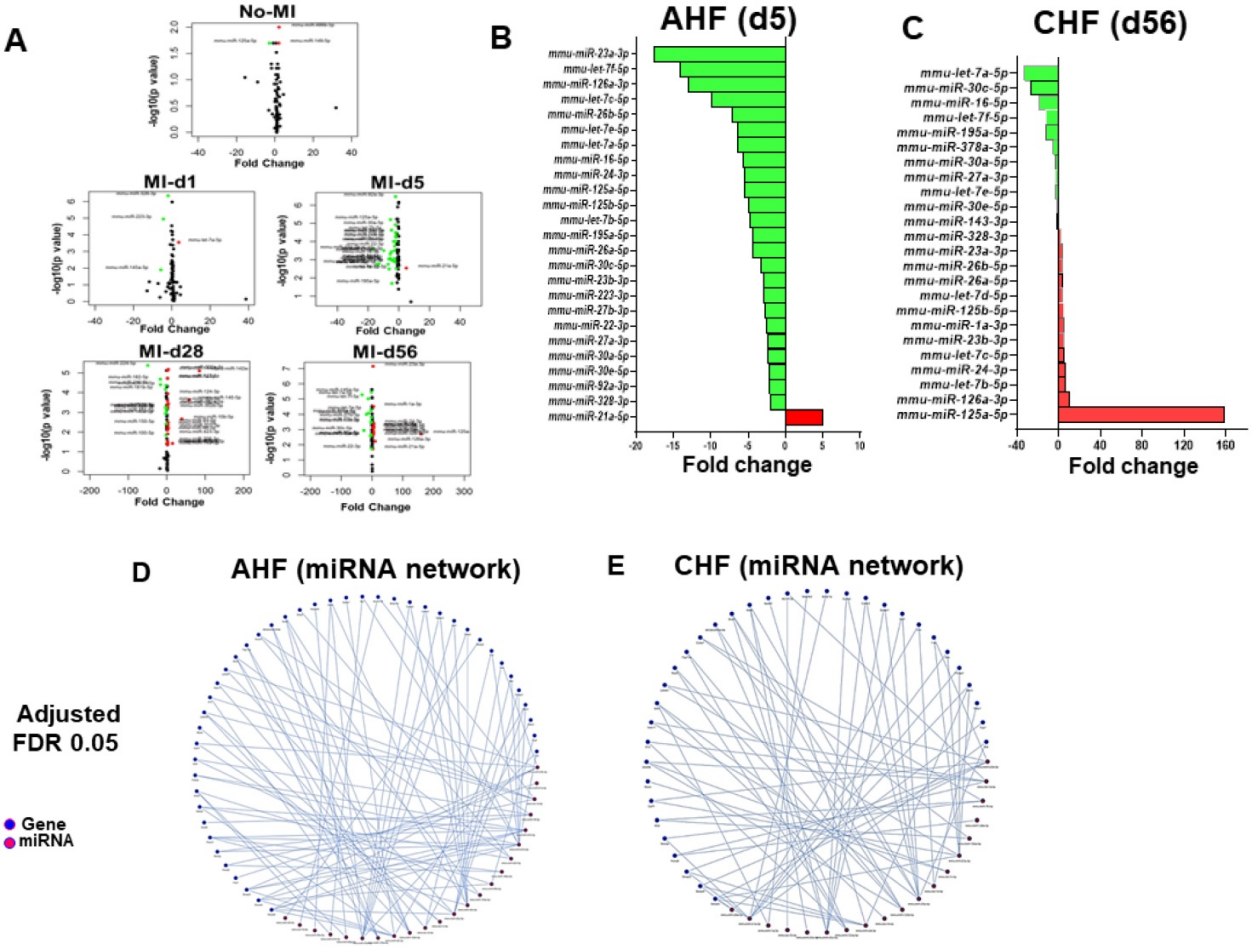
** Comprehensive miR regulation in 12/15LOX null mice during AHF and CHF. (A)** Volcano plot for miRs in AHF and CHF in 12/15LOX^-/-^ mice respective to the WT controls. The X axis is log2 fold-change, and the Y axis is log fold change. In graphs, each point represents an individual transcript. The up-regulated (right, red dots) and down-regulated (left, green dots) miRs are shown. **(B)** Bar graph representing fold change of miRNA in 12/15LOX^-/-^ mice at AHF. **(C)** Bar graph representing fold change of miRNA in 12/15LOX^-/-^ mice at CHF. **(D, E)** 121/5LOX gene interaction network generated through Mienturnet_network. The cutoff is 2-fold change (n = 4 mice/group for each time point). Student t-test (unpaired) was applied comparing miR exression at the particular day to naive control(d0).

Ample of evidence suggest that array of miRs have direct role in cytokines and chemokine mRNAs activation and is widely involved in cardiac inflammation program 21. To further understand whether amplified cardiac repair in 12/15LOX mice is associated with change in miRs, we analyzed a set of miRs in AHF and CHF. Comprehensive miRs analysis of 84 miRNA panel presented as volcano plot indicates that in 12/15LOX null mice 5 miRNA differentially regulated in no-MI controls. During AHF (MI-d1), only 4 miRs significantly changed because of 12/15LOX deletion. However, numbers of miRs were differentially changed in infarct heart during AHF (post MI-d5) and in CHF (post MI-d28 and d56) **(Figure 4A and Figure S6)**. A total of 26 miRs related to fibrotic remodeling were significantly impacted at d5 post-MI, out which only miR-21a-5p was upregulated by 5 fold and 25 miRs was significantly downregulated~4 fold (miR-214-3p, miR-328-3p, miR-92a-3p, miR-30e-5p, miR-30a-5p, miR-27a-3p, miR-22-3p, miR-27b-3p, miR-223-3p, miR-23b-3p, miR-30c-5p, miR-26a-5p, miR-195a-5p, let-7b-5p, miR-125b-5p, miR-125a-5p, miR-24-3p, miR-16-5p,let-7a-5p, let-7e-5p, miR-26b-5p,let-7c-5p, miR-126a-3p, let-7f-5p, miR-23a-3p) suggested resolving response **(Figure 4B; Table S1)**. During, progressive HF (post-MI d28) an equivalent response miRs was observed, in which 18 miRs were significantly up-regulated, and 11 were significantly down-regulated **(Table S1)**. Deficiency of 12/15LOX normalized these changes in CHF (post-MI d56) while 13 miRNAs were upregulated and 11 were down regulated. In CHF, 100-fold upregulation in miR-125a-5p strongly suggested MΦ polarization towards resolving phenotype **(Figure 4C; Table S2)**. The Mienturnet network analysis revealed differentially regulated miRs that are related to extracellular matrix remodeling, glucose metabolism, and cytokines **(Figure 4D-E)**. Our miR array data outcome suggested that 12/15LOX deletion modulates differential miRs counter inflammation and facilitates resolving phenotype of MΦs in cardiac repair.

### 12/15LOX deletion limited the MI-induced cardio-renal inflammation and delayed the transition of acute to chronic HF

**Figure 5:**
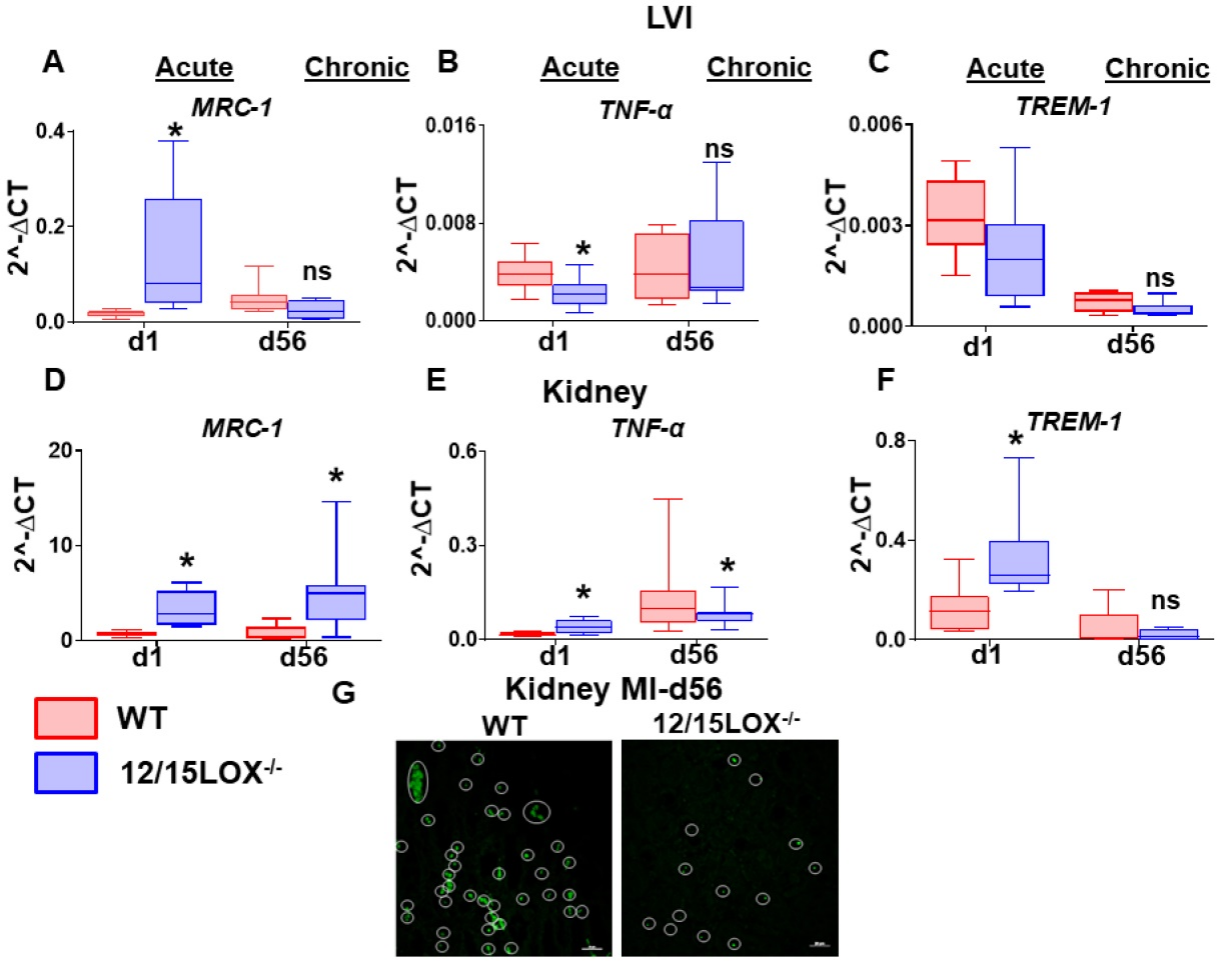
** 12/15LOX deletion modulates MI-induced cardiorenal pro-resolving and pro-inflammatory response in AHF and CHF.** Kidney and LVI mRNA expression of **(A, D)** proresolving marker-*MRC-1* **(B, E)** proinflammatory marker-*TNF-α* **(C, F)** Monocyte/macrophage- and neutrophil-mediated inflammatory response marker-*TREM-1* at acute (post-MI d1) and chronic (post-MI d56) phase in WT and 12/15LOX^-/-^ mice. Values are represented as mean ± SEM; n = 5 *p < 0.05 vs WT. Analysis of variance (ANOVA), followed by Newman-Keuls post-hoc test, was used for multiple comparisons. (G) TUNEL staining at post-MI d56 in kidney representing decrease in apoptotic cells (TUNEL positive cell: green) in 12/15LOX^-/-^ mice compared with WT mice (magnification 40X, scale = 20µm). Images are representative of 5-8 field/slide; n = 4 slide/group.

At organism level, the heart and kidney are irrevocably linked 23. As such, this inter-dependency of heart and kidney results in a vicious circle where the dysfunctional heart can impact the kidney or vice versa as a cardiorenal/reno-cardiac syndrome. Thus, we further investigated the levels of proinflammatory and proresolving markers in transition of AHF to CHF in LV and kidney simultaneously. The mRNA expression of *Mrc-1* was upregulated at d1 (acute) in both LVI (4.7 -fold, p < 0.05) and kidney (5.0 -fold, p < 0.05) in 12/15LOX^-/-^ mice compared with WT. During CHF (d56) the *Mrc-1* remain elevated (9.0 -fold, p < 0.05) in 12/15LOX^-/-^ compared with WT in LVI **(Figure 5A and 5D)**. However, *Mrc-1* expression was decreased in kidney in CHF compared with the acute phase with no difference in the levels of expression between WT and 12/15LOX^-/-^. Pro-inflammatory cytokine such as *TNF-α* was upregulated (p < 0.05) in 12/15LOX^-/-^ LVI compared with WT during the acute phase with no significant difference at d56 suggestive of complete resolution phase. The kidney displayed lower levels of *TNF-α* in 12/15LOX^-/-^ mice compared to WT during the acute phase. In CHF, *TNF-α* levels were upregulated with no significant difference between WT and 12/15LOX^-/-^ kidneys **(Figure 5B and 5E)**. To determine, early impact on immune response due to ischemia, we determined the expression of innate immune system amplifier *Trem-1(Triggering receptor expressed on myeloid cells 1)* in both LV and kidney. *Trem-1* levels were observed to follow opposite trends in LV infarct and kidney during the acute phase in 12/15LOX^-/-^ mice compared with WT. The levels of *Trem-1* were upregulated in 12/15LOX^-/-^ mice compared with WT in LV and vice versa in the kidney. In CHF, the *Trem-1* was downregulated with no significant difference in LV and kidney **(Figure 5C and 5F)**. To confirm the MI-induced apoptosis in kidney during in CHF, the kidney stained using TUNEL methodology particularly in CHF. The representative kidney (medulla) section shows higher number of apoptotic nuclei in WT kidneys were compared with 12/15LOX^-/-^ kidneys **(Figure 5G)**. Thus, our results suggest 12/15LOX deletion not only counter inflammatory response in acute HF but also resolves renal inflammation in CHF.

### Abrogation of 12/15LOX suppressed the MI-induced kidney injury in progressive HF

**Figure 6:**
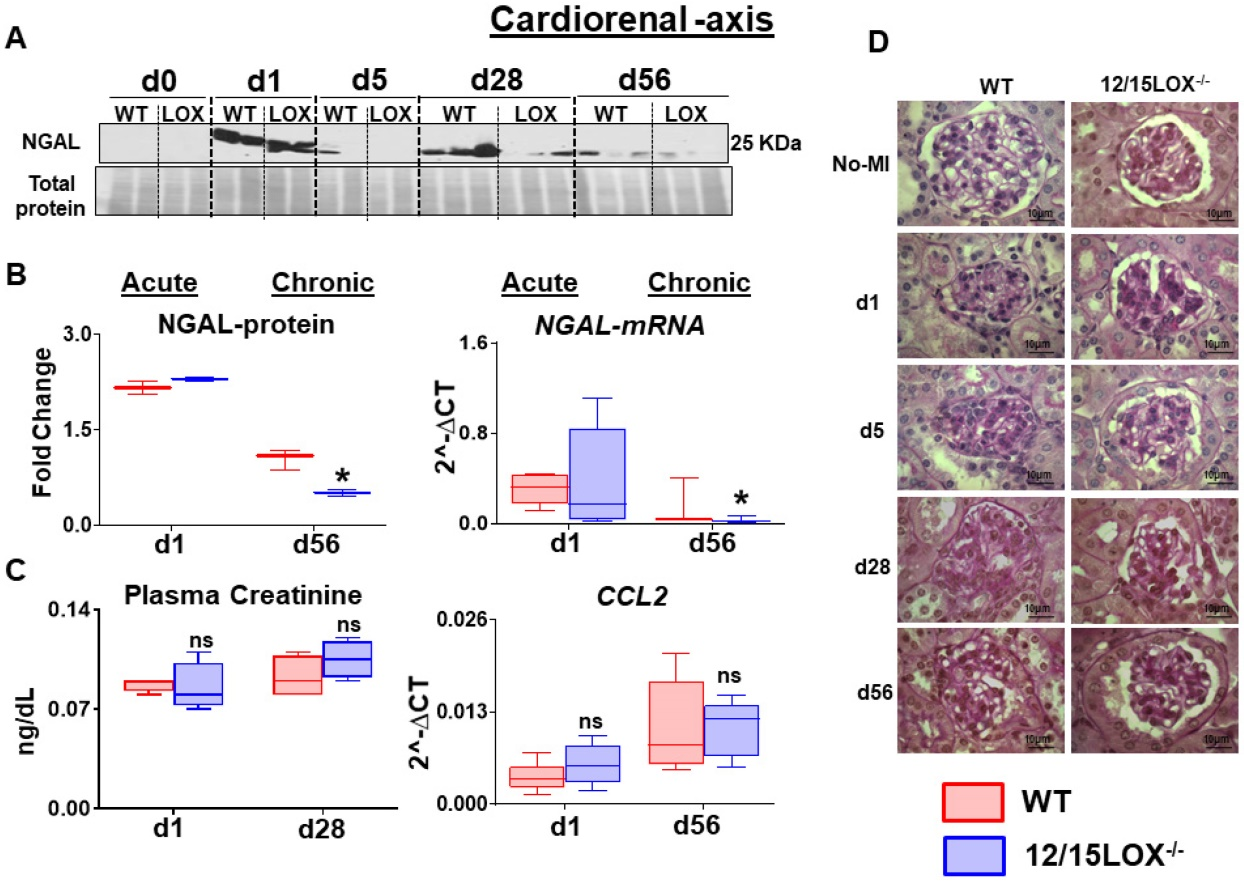
** 12/15LOX^-/-^ mice have limited MI-induced kidney injury and apoptosis in progression of heart failure. (A)** Immunoblot representing decrease expression of NGAL at post MI d28 and d56 in 12/15 LOX^-/-^ mice compared with WT. **(B)** Densitometric analysis and mRNA expression of NGAL at acute (post-MI d1) and chronic (post-MI d56) phase in WT and 12/15LOX^-/-^ mice. **(C)** Plasma creatinine levels measured by mass spectroscopy and mRNA expression of *ccl2* at acute (post-MI d1) and chronic (post-MI d56) phase in WT and 12/15LOX^-/-^ mice. Values are represented as Whisker and box plots showing median and interquartile range. Minimum and maximum values are represented in each group by the whiskers of the plot; n = 5 mice *p < 0.05 vs d0 respective control, # *p* < 0.05 vs WT at respective time point. Analysis of variance (ANOVA), followed by Newman-Keuls post-hoc test, was used for multiple comparisons. **(D)** PAS staining in kidney in No-MI and from post-MI d1 to d56 showing glomerulus in WT and 12/15LOX^-/-^ mice (magnification = 100X). Images are representative of 4 - 5 field/slide; n = 4 slide/group.

After ischemic insult, the renal inflammation is immediately caused by acute LV injury. Thus, renal inflammation determined during acute and chronic HF stages between WT and 12/15LOX^-/-^ mice. Post-MI renal inflammation was confirmed by the early upregulation of neutrophil gelatinase-associated lipocalin (NGAL) at the acute phase in the kidney. Both WT and 12/15LOX^-/-^ mice displayed a similar level of NGAL expression during the acute phase. In CHF, the 12/15LOX^-/-^ mice limited NGAL expression at d1 and d5 post-MI compared with their respective MI-control in progression of HF when compared with WT **(Figure 6A-B)**. However, there was no significant difference in creatinine and CCL2 levels between WT and 12/15LOX^-/-^ during the acute and chronic phases **(Figure 6C)**. Renal microstructure assessed by histology displayed limited mesangial expansion in glomeruli suggesting reduced glomerulonephritis in 12/15LOX^-/-^ mice during the progression of HF (d56) compared with WT mice **(Figure 6D)**. Thus, early, and late kidney injury inflammatory markers indicated that MI-induced cardiorenal inflammation limited in 12/5LOX^-/-^ mice in CHF post-MI.

## Discussion

In response to myocardial injury and subsequent repair, infiltrating leukocytes are prime infiltrating cells that orchestrate inflammatory-resolving and reparative signaling. Traditionally, resolution after initiation of inflammation was consider to be a passive event, however with current prevalent reports particularly after heart attack confirms that inflammation coincides with resolution as an active event that facilitates cardiac repair 5, 24. Pre-clinical studies that are focused on short-term window of initiation, inflammation, and resolution (safe clearance of inflammation) is critical between physiological cardiac repair (physiological inflammation) and pathological remodeling that defines the trajectory of advanced HF. In cardiac repair, LOX enzymes catalyze metabolic transformation of fatty acids into class-switching of lipid mediators and interaction with the specific type of fatty acid that determines the leukocyte inflammatory-reparative phenotypes 9, 25. Our previous report indicates that 12/15LOX deletion improves post-MI survival and heart function with increased levels of EETs in the heart and spleen, thereby promoting effective resolution in AHF 9, 10. In the current study, we discovered novel mechanism that provides the basis of cardiac repair after 12/15LOX deletion: 1) increased EP4 expression in reparative MΦ during acute phase; 2) upregulation of miR23a in infarcted heart facilitating cardiac repair; 3) increase in post-MI survival and LV function during CHF; and 4) decreased in cardiorenal inflammation during CHF. Thus, our data provides an evidence that 12/15LOX deficiency activates EP4 on MΦs during the second wave of the bimodal response thereby delaying progression of HF and limits cardiorenal inflammation (graphical abstract).

### Activation of EP4 and reparative MΦ in cardiac repair

Interaction of prostaglandin E2 (PGE2) receptor and respective 4 or E-type prostanoid receptors (EP4) are known to play a multiple role in cardiac remodeling 26. Overexpression of EP4 improves cardiac function post-MI, while deficiency of EP4 receptor in cardiomyocytes (EP4-KO) have worsened heart function post-MI and develops age-related dilated cardiomyopathy 27-29. In contrast, a study has stated PGE2 activates cardiomyocyte EP4 to induce hypertrophy, 30 however it is unclear whether this hypertrophy is adaptive or maladaptive. The studies in mice have also reported that the depletion of EP2 significantly impaired cardiac repair due to failed initiation of MΦ recruitment to the injured myocardium 31. Our data suggest that both EP2 and EP4 were expressed in AHF and CHF and have distinct signaling in different tissues. Both EP2 and EP4 are amplified in AHF (d1 and d5) post-MI, however EP2 expression diminishes in CHF with no change in the LV of WT or 12/15LOX^ -/-^ mice. During cardiac repair, robust increase of EP4 expression during AHF and CHF validates reparative role due to precise localization in infarcted zone of post-MI hearts indicative of expedited healing and hastened repair in 12/15LOX^-/-^ mice. Present results align with the previous findings that suggest deficiency of EP4 increased infarct size post-I/R injury when compared with control mice. Likewise, pharmacological intervention with an EP4 agonist reduced infarct size and improved cardiac function 32 , 33. MΦs are known to have abundant EP4 expression 34, thus EP4 activation is involved in ameliorating chronic inflammatory diseases 35. Staining specificity of EP4 expression confirmed the presence on NΦs, MΦs, and CFs, particularly *in vitro* study confirmed EP4 is highly expressed on proresolving MΦ2. *In vitro* results concord with previous reports that suggest 12/15LOX null mice have higher M2 (pro-resolving MΦ) and increased survival compared with WT due to increased levels of epoxyeicosatrienoic acids (EETs) in spleen and heart 5, 9. We recently demonstrated that female (C57BL/6) mice have higher levels of EETs and survives better than their male counterpart 36. Yang et al has shown that EETs, specifically 14,15-EET could mimic PGE_2_ in rat mesenteric arteries 37. PGE_2_ is one of the most abundant species with an important role in both initiation and resolution of inflammation because the failure of initiation obviously delays the resolution thereby impaired cardiac repair. Higher expression of EP4 on MΦs, defines the role of resolving MΦs phenotype in cardiac repair.

### miRs polarize MΦ phenotype in initiation and resolution of inflammation

miRs silently tailors the immune system which regulates inflammation and cardiac remodeling 38-40. 12/15LOX deletion influenced broad range of miRs which regulates cardiac hypertrophy, regulatory cytokines, immune-responsive genes which are critical in cardiac remodeling and transition from AHF to CHF 21. Though 12/15LOX deletion influenced few miRs in naïve control but during the early acute phase (MI-d5), down regulation of several miRs was noted. Of these, miR-23a-3p is significantly downregulated (almost 20-fold). Particularly, miR-23a-3p cluster can regulate inflammatory (M1) and reparative (M2) cytokines and in turn could simultaneously regulate MΦ polarization through a negative feedback and feedforward loop 41, 42. Of note, down regulation miR-23a-3p in 12/15LOX^-/-^ mice with limited cardiac remodeling is due to a switch in M1 to M2 suggesting a shift towards reparative signaling. This further continued in CHF with upregulation of miR-125a-5p about 160-fold which enhances alternative activation of MΦ dampening inflammatory response. Present results suggest that 12/15LOX deletion differentially adapts both negative and positive feedback loop to regulate miRs in AHF and CHF, respectively.

### Acute survival defines long-term heart function and CHF survival

Previous report indicated that 12/15LOX deficiency improves post-MI survival (monitored till day 28). This clearly supports previous findings that are further extended to day 56 as an advanced HF 9. Present report validates the survival and the bimodal response of 12/15LOX^-/-^, which not only improved post-MI survival but also improved heart function and efficiency of resolving inflammation, which is critical for transition from AHF to CHF. Though the current study displayed increased survival, it should be taken into account that these mice do not have any cofounding or co-existing comorbid factors (such aging or metabolic defects) that are commonly seen in the HF patients.

### Resolution of cardiorenal inflammation

Renal dysfunction is a common comorbidity in advanced or end stage HF patient with diminished kidney function 4. This co-occurrence of cardiac and kidney diseases are progressive and bidirectional two-hit stress that may explain multi-organ dysfunction 43. Thus, clearance and on time control of inflammation limit the pathogenesis of both cardiovascular and renal diseases. Given the circulating nature of pro-inflammatory mediators (cytokines, immune cells) and leukocyte-derived bioactive lipids, it is speculated that the immune system can act as a mediator of organ crosstalk. Immune cells may be involved in the reciprocal dysfunction that is encountered commonly and distinctly behave between acute versus chronic HF settings. Decoding of cytokines and lipid mediators profile revealed that the different cytokine/lipid mediators respond differently in acute and CHF in LV and kidney. The former can be defended by expression of *TREM-1* which stimulates neutrophil and monocyte-mediated inflammatory responses were higher during the AHF but *TNF-α* was elevated in kidney in CHF. *NGAL* is a potent acute kidney injury biomarker, which is immediately elevated within 24h post-MI as a result of the acute inflammatory in heart and kidney 44. Even though in AHF, 12/15LOX null mice showed no difference between in NGAL but a significant decrease of protein expression in CHF indicative of reduced renal inflammation.

## Limitations

In the presented manuscript we used risk-free young-adult mice, consideration of risk factor such as aging or co-medication or co-morbidity like metabolic syndrome model or use of high fat diet intervention, co-medication(s), the expected outcome may be different which is not covered in this manuscript 4, 45.

In summary, our study uncovered the mechanism that 12/15LOX deficiency polarize the MΦ towards reparative phenotype with activation of EP4 receptor during AHF, which swiftly resolves inflammation and increases survival and heart function in progressive HF. Therefore, 12/15LOX could be potential target for cardiovascular medicine in transition of AHF to CHF.

## Supplementary Material

Supplementary figures.

Supplementary tables.

## Figures and Tables

**Table 1:** Echocardiography and necropsy results in WT and 12/15LOX^-/-^ heart failure survivor compared to naïve controls

	No-MI		MI d1		MI d5		MI d28		MI d56	
	WT	12/15LOX^-/-^	WT	12/15LOX^-/-^	WT	12/15LOX^-/-^	WT	12/15LOX^-/-^	WT	12/15LOX^-/-^
n	7	5	4	5	4	7	8	6	5	10
Heart Rate (bpm)	452±13	427±9	439±7	448±23	511±11	428±12	443±17	428±9	440±21	409±7
EDD (mm)	3.60±0.1	3.59±0.03	4.91±0.3*	4.76±0.2*	5.65±0.4*	5.00±0.2*^$^	5.75±0.2*	5.34±0.1*^$^	5.83±0.4*	5.42±0.2*^$^
ESD (mm)	2.48±.18	2.33±.07	4.60±.29	4.36±.22	5.35±.44	4.61±.16	5.42±.27	4.91±.12	5.53±.41	4.98±.21
Fractional Shortening %	35±2	35±1	6.3±0.5*	8.4±1*^$^	5.5±1*	7.7±0.7*^$^	5.9±1*	8±0.6*^$^	5.2±0.7*	8.1±0.5*^$^
PWTs (mm)	1.01±.06	1.03±.02	0.53±.06*	0.46±.03*	0.43±.06*	0.51±.03*^$^	0.40±.03*	0.52±.07*^$^	0.33±.06*	0.44±.03*
EDV (**µ**l)	64±5	44±2	88±7*	80±4*	92±9*	90±3*	128±15*	114±6*	135±12*	112±14*^$^
ESV (**µ**l)	23±3	14±1	76±6*	65±5*	82±9*	77±3*	111±14*	97±6*	119±24*	92±13*
Ejection Fraction	67±2	65±3	14±1*	19±3*	12±2*	15±1*	14±2*	15±2*	13±2*	19±2*^$^
Body weight (g)	23.43±0.8	23.5±1.1	20.43±0.3	22.98±1.2	22.50±1	23.51±0.6	26.50±0.5	23.44±1.1	26.52±0.4	25.75±0.4
LV (mg)	72.3±1.9	74.4±3.8	74.3±1.5	81.8±5.6*	98.8±0.9*	86.3±1.4*^$^	108.3±6.1*	91.3±7.4*^$^	100.0±6.6*	100.9±5.38
LV/BW (mg/g)	3.1±0.02	3.2±0.08	3.6±0.07*	3.6±0.08*	4.4±0.2*	3.7±.01*^$^	4.0±0.2*	3.8±0.1*^$^	3.8±0.2*	3.9±0.1*
RV (mg)	17±1	25±2*	13±1	20±3	22±2	20±1	25±1	21±1	23±2	23±1
Lung weight	119±10	127±8	138±27*	128±4	180±29*	127±4^$^	141±1*	137±12	132±13*	149±17*^$^
Infarct Area (%)	n/a	n/a	49.3±1.6*	47.5±2.3*	52.5±1.6*	50.1±1.9*	52.4±1.5*	52.1±1.4*	53.1±1.2*	51.9±2.1*
RV mass/BW	0.66±0.2	1.06±0.1*	0.65±0.04	0.87±0.1*^$^	0.96±0.1*	0.85±0.1*^$^	0.93±0.02*	0.90±0.1*	0.88±0.1*	0.91±0.1*^$^

Values are mean± SEM; n indicates sample size. bpm: beats per minute; EDD: end-diastolic dimension; ESD: end-systolic dimension; PWT: Infarcted wall thickness, S: systole; mm: millimeter; LV: Left Ventricular; BW: Body weight; RV: Right Ventricular; ND: not detectable, *p < 0.05 vs. day 0 wild-type (WT) control; $*p* < 0.05 vs. WT with respective time point.
