# Supplementary material for: Activation of EP4 receptor limits transition of acute to chronic heart failure in lipoxygenase deficient mice
Source: Theranostics. 2021 Jan 1;11(6):2742–54. doi: 10.7150/thno.51183 (PMC7806484; doi:10.7150/thno.51183)
Supplement: Supplementary file 1 — Supplementary figures. [file thnov11p2742s1.pdf]

Supplementary figure legends

Figure-S1:

Plane polarized light applied LV-mid-cavity images stained with picro sirius red indicative of fibrosis.

(A) 1.25x images of the complete LV. (**B)** 40x images of the LV infarct zone. **(C)** 40X images of the LV peri-infarct zone (border) in WT and 12/15LOX$^{-/-}$ mice during CHF (post-MI d28 and d56) displaying changes in fibrosis (scale bar = 50 µm); n = 10 mice/group/day.

Figure-S2:

12/15LOX deletion robustly increases EP4 not EP2 in LV infract post-MI in progressive heart failure with monocyte trafficking marker CCL2.

(A) Immunoblot showing robust increase EP2 and CCL2 in post-MI in WT and 12/15LOX$^{-/-}$ mice in heart failure. **(B)** mRNA expression of *EP2* **(C)** Densitometric analysis of EP2 in WT and 12/15LOX$^{-/-}$ mice in no-MI and post-MI d1 till d56**. (D)** mRNA expression of EP2 in isolated leukocytes at post-MI d1, d5 and in cardiac fibroblast (CF) at post-MI d5 isolated from LV infarct. **(E)** mRNA expression of *EP2* on peritoneal MΦ (Naïve, M1 and M2 phenotype) isolated from WT and 12/15LOX$^{-/-}$ mice. **(G)** mRNA expression of *CCL2* **(H)** Densitometric analysis of *CCL2* in WT and 12/15LOX$^{-/-}$ mice in no-MI and post-MI d1 till d56. Box plots show median and interquartile range. Minimum and maximum values are represented in each group by the whiskers of the plot; n = 2 – 5 mice/group/day; * $p < 0.05$ vs d0 respective control, # $p < 0.05$ vs WT at respective time point. Analysis of variance (ANOVA), followed by Newman–Keuls post-hoc test, was used for multiple comparisons.

Figure-S3:

Expanded representative immunofluorescence images displaying images cardiomyocyte area by WGA staining (green) and EP4 expression (red) in LV infarct and LV remote are of WT and 12/15LOX$^{-/-}$ mice in no-MI controls and at d5 post MI (magnification= 40X, scale =20 µm). Images are representative of 5 - 8 field/slide; n = 4 slide/group

Figure-S4:

Expanded representative immunofluorescence images displaying EP4 expression (red) and nuclei (blue) on peritoneal macrophages (Naïve, M1 and M2 phenotype) isolated from WT and 12/15LOX$^{-/-}$ mice. WT peritoneal macrophages (Naïve, M1 and M2 phenotype) was treated with EP4 agonist

(CAY10598 - 100nM) to confirm EP4 stimulation (magnification = 40X, scale = 50 µm; zoom = 5.72; scale =10 µm). Images are representative of 5 - 8 field/slide; n = 4 slide/group.

Figure-S5:

Expanded representative immunofluorescence images displaying EP4 expression (red) and nuclei(blue) on cardiac fibroblast and myofibroblast (treated with TGF-β-15 ng/mL for 18 h) isolated from WT and 12/15LOX$^{-/-}$ mice (magnification = 40X, scale = 50 µm; zoom = 3.53; scale = 10 µm). Images are representative of 5 - 8 field/slide; n = 4 slide/group.

Figure S6:

Bar graph representing fold change of statistically significant miRNAs in 12/15LOX$^{-/-}$ mice in progression of AHF to CHF. (The cut off is 2 fold change); n = 4 mice/group for each time point. Student t-test (unpaired) was applied comparing miRs expression at the particular day to naive control (d0).

S Figure 1

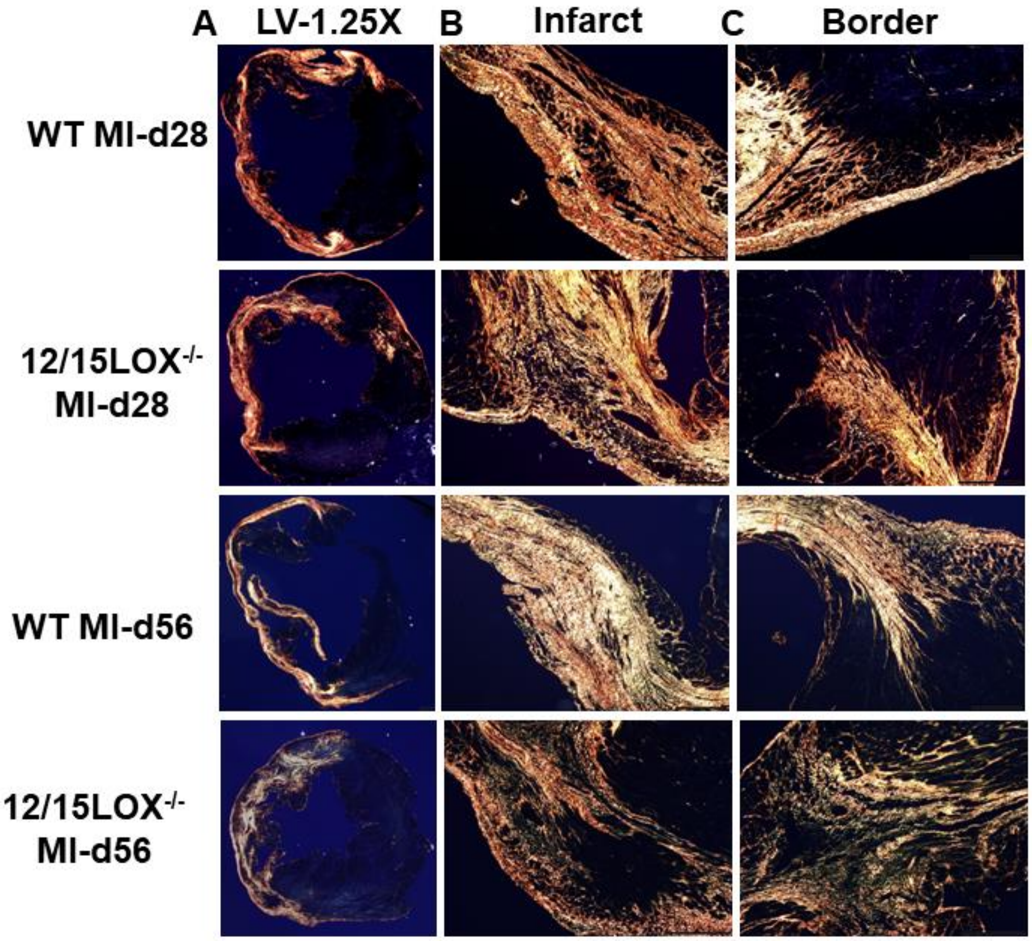
A
LV-1.25X
B
Infarct
C
Border
WT MI-d28
12/15LOX-/-
MI-d28
WT MI-d56
12/15LOX-/-
MI-d56

S Figure 2

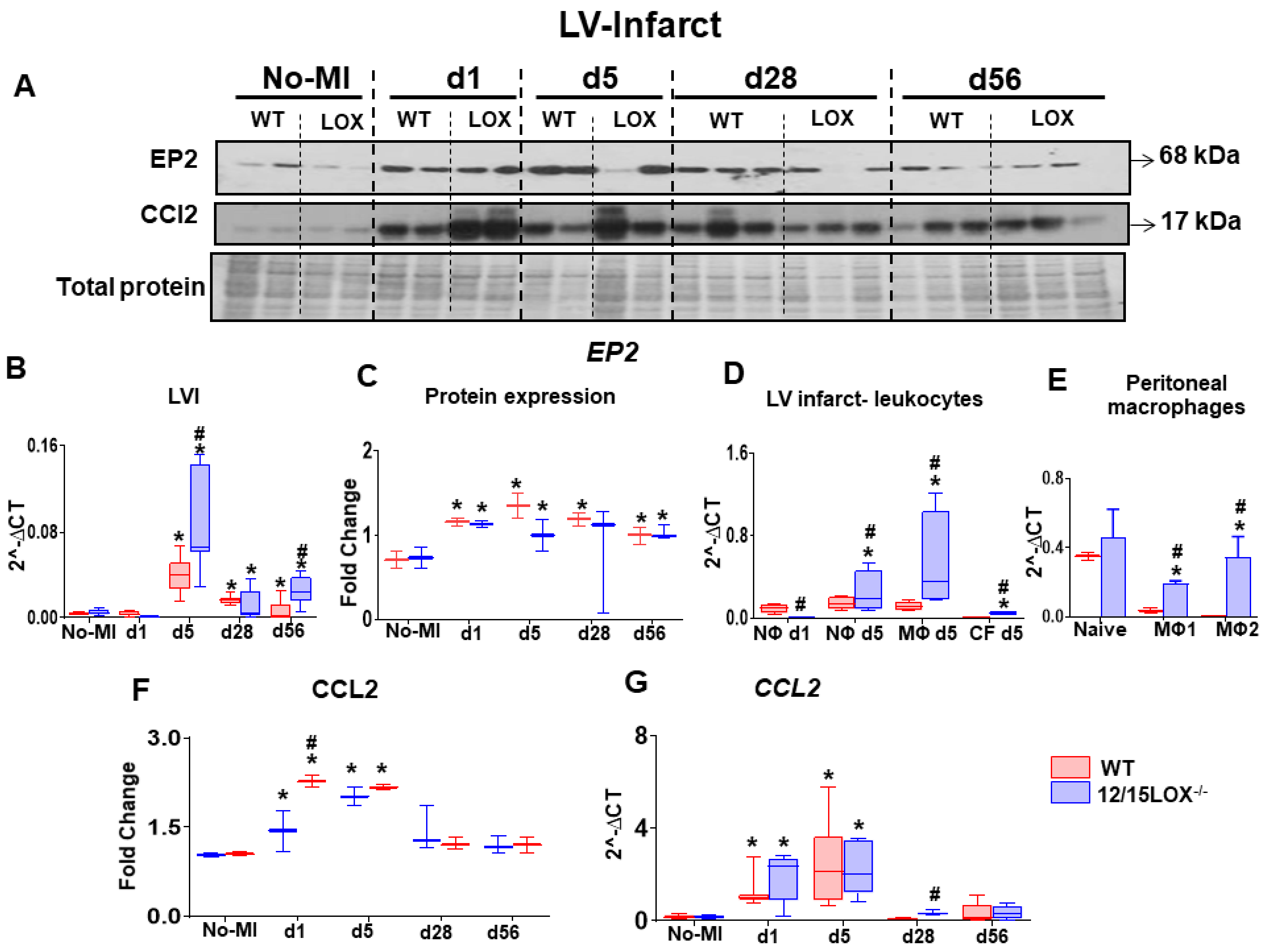
LV-Infarct
A
No-MI
d1
d5
d28
d56
WT
LOX
EP2
CCl2
Total protein
68 kDa
17 kDa
EP2
B
LVI
2^-ΔCT
No-MI d1 d5 d28 d56
C
Protein expression
Fold Change
No-MI d1 d5 d28 d56
D
LV infarct- leukocytes
2^-ΔCT
NΦ d1 NΦ d5 MΦ d5 CF d5
E
Peritoneal macrophages
2^-ΔCT
Naive MΦ1 MΦ2
F
CCL2
Fold Change
No-MI d1 d5 d28 d56
G
CCL2
2^-ΔCT
No-MI d1 d5 d28 d56
WT
12/15LOX-/-

S Figure 3

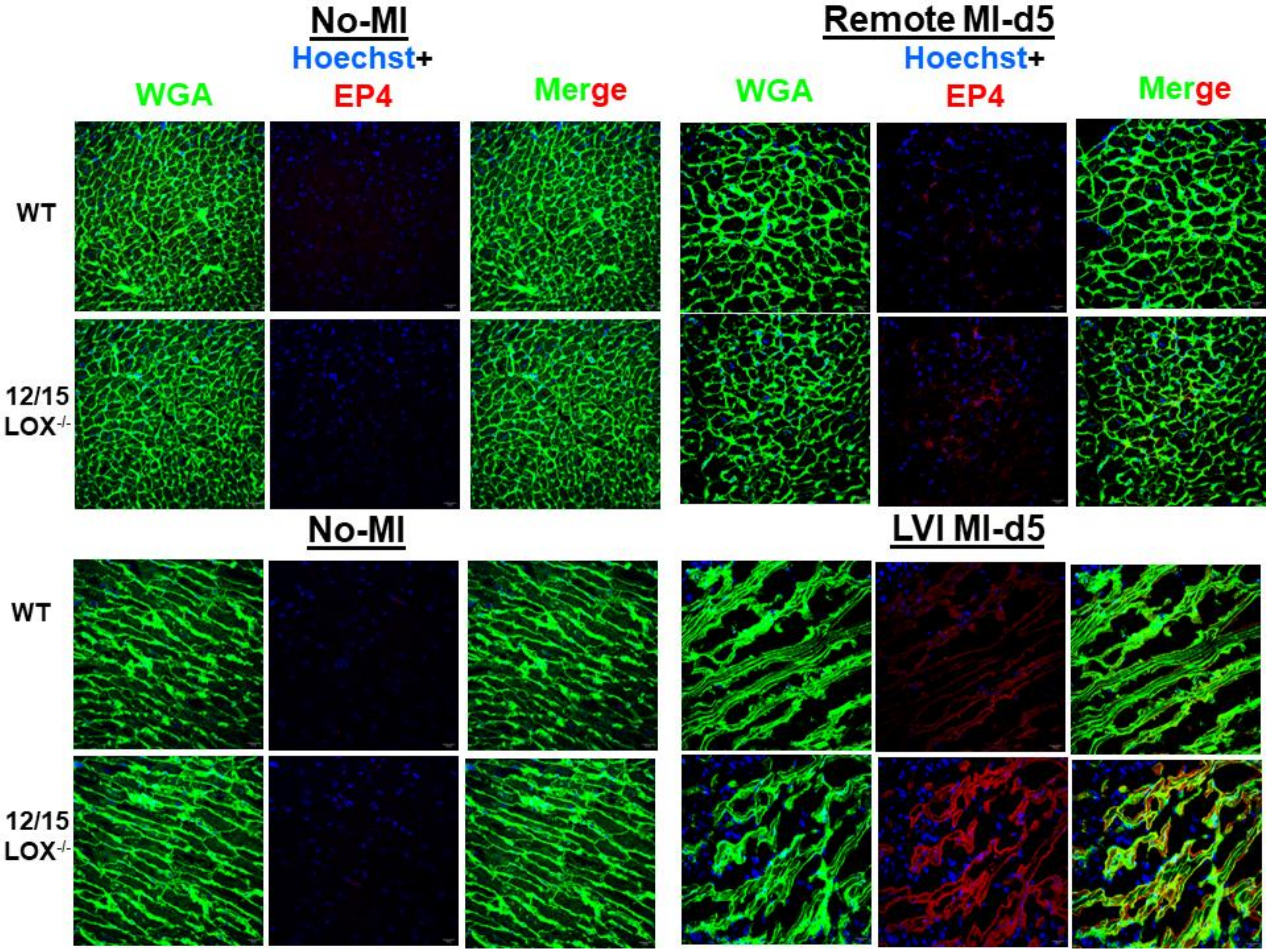
No-MI
Remote MI-d5
WGA
Hoechst+
EP4
Merge
WGA
Hoechst+
EP4
Merge
WT
12/15 LOX-/-
No-MI
LVI MI-d5
WT
12/15 LOX-/-

S Figure 4

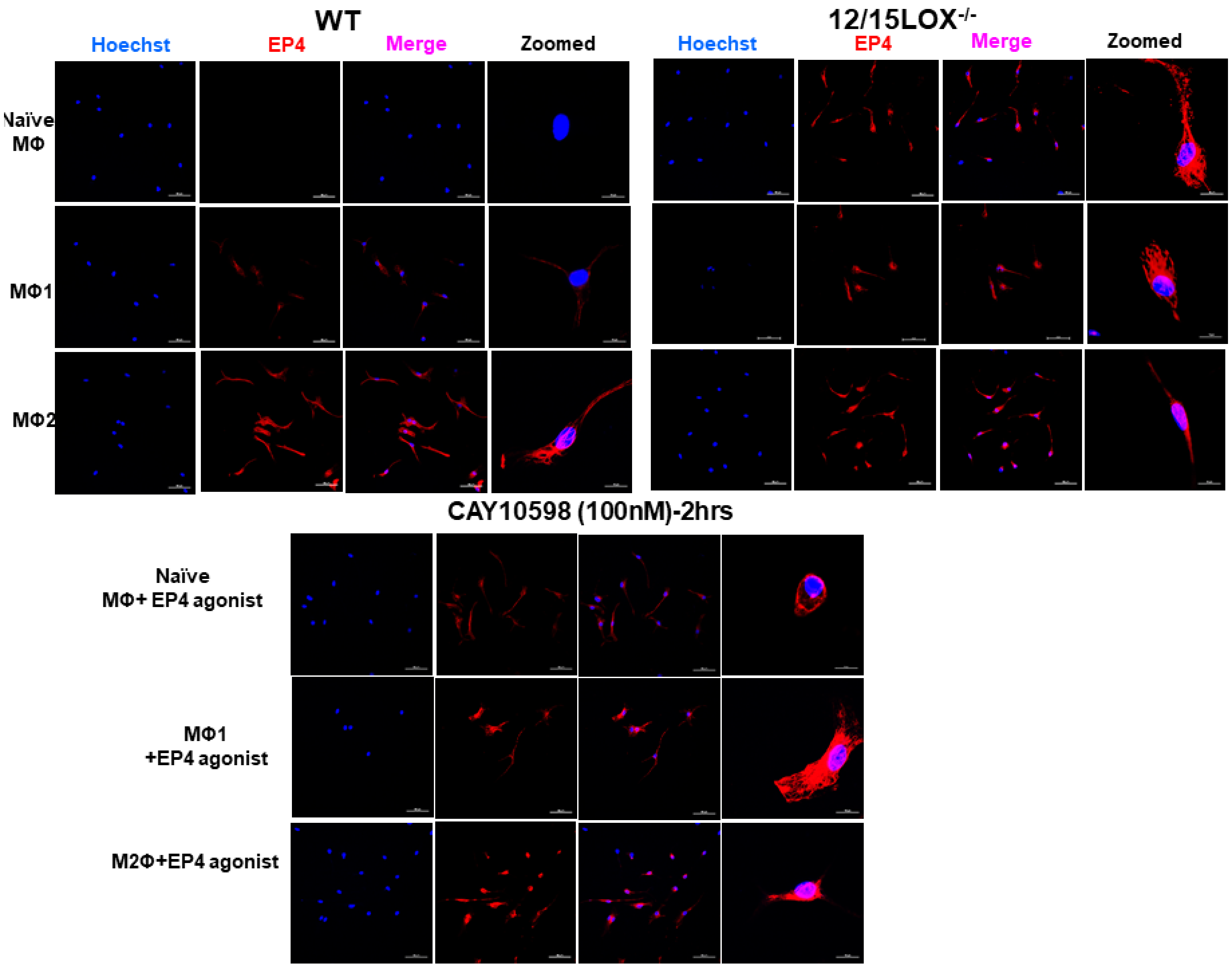
WT
Hoechst
EP4
Merge
Zoomed
12/15LOX-/-
Hoechst
EP4
Merge
Zoomed
Naïve MΦ
MΦ1
MΦ2
CAY10598 (100nM)-2hrs
Naïve MΦ+ EP4 agonist
MΦ1 +EP4 agonist
M2Φ+EP4 agonist

S Figure 5

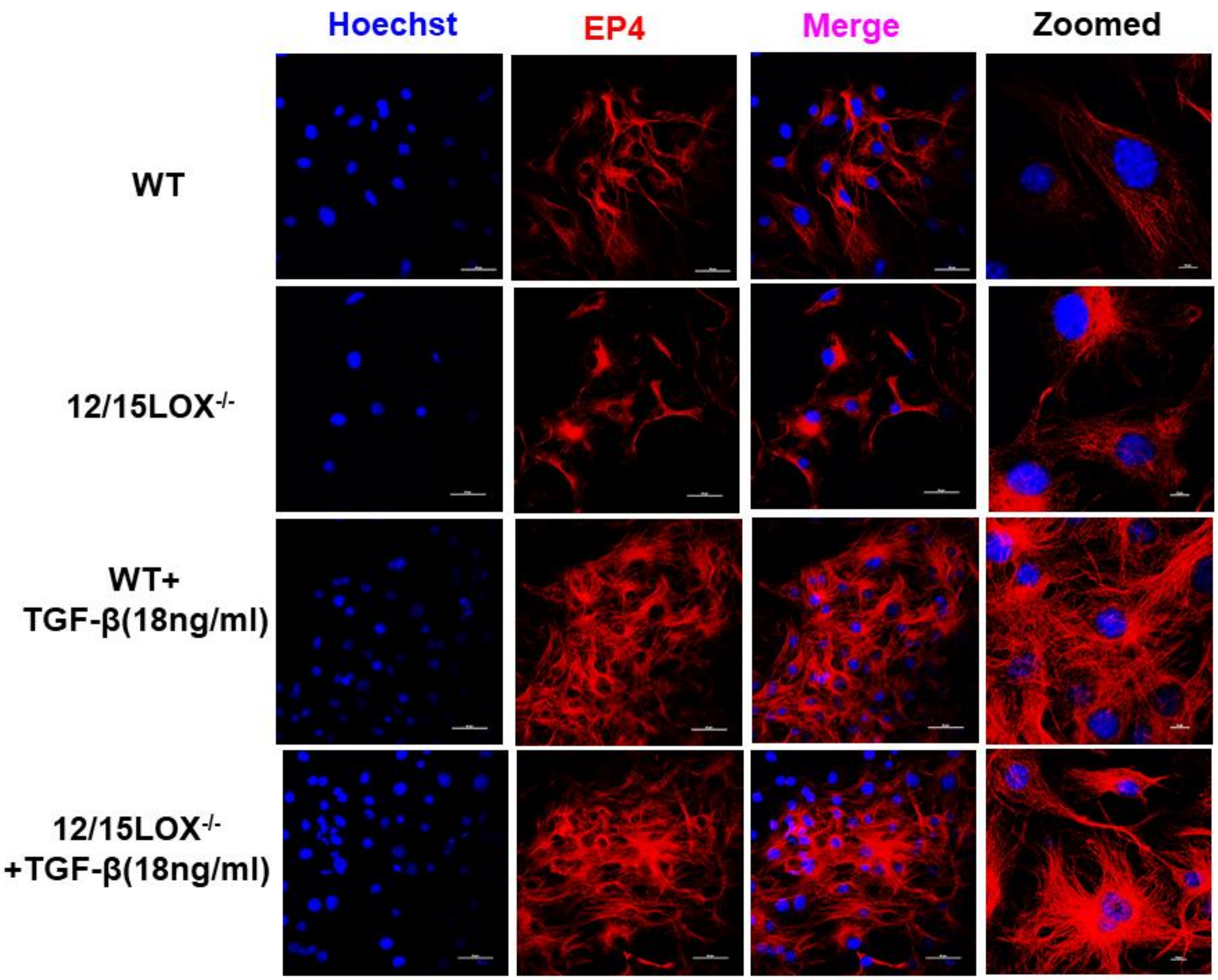
Hoechst
EP4
Merge
Zoomed
WT
12/15LOX-/-
WT+
TGF-β(18ng/ml)
12/15LOX-/-
+TGF-β(18ng/ml)

1 **S Figure 7**

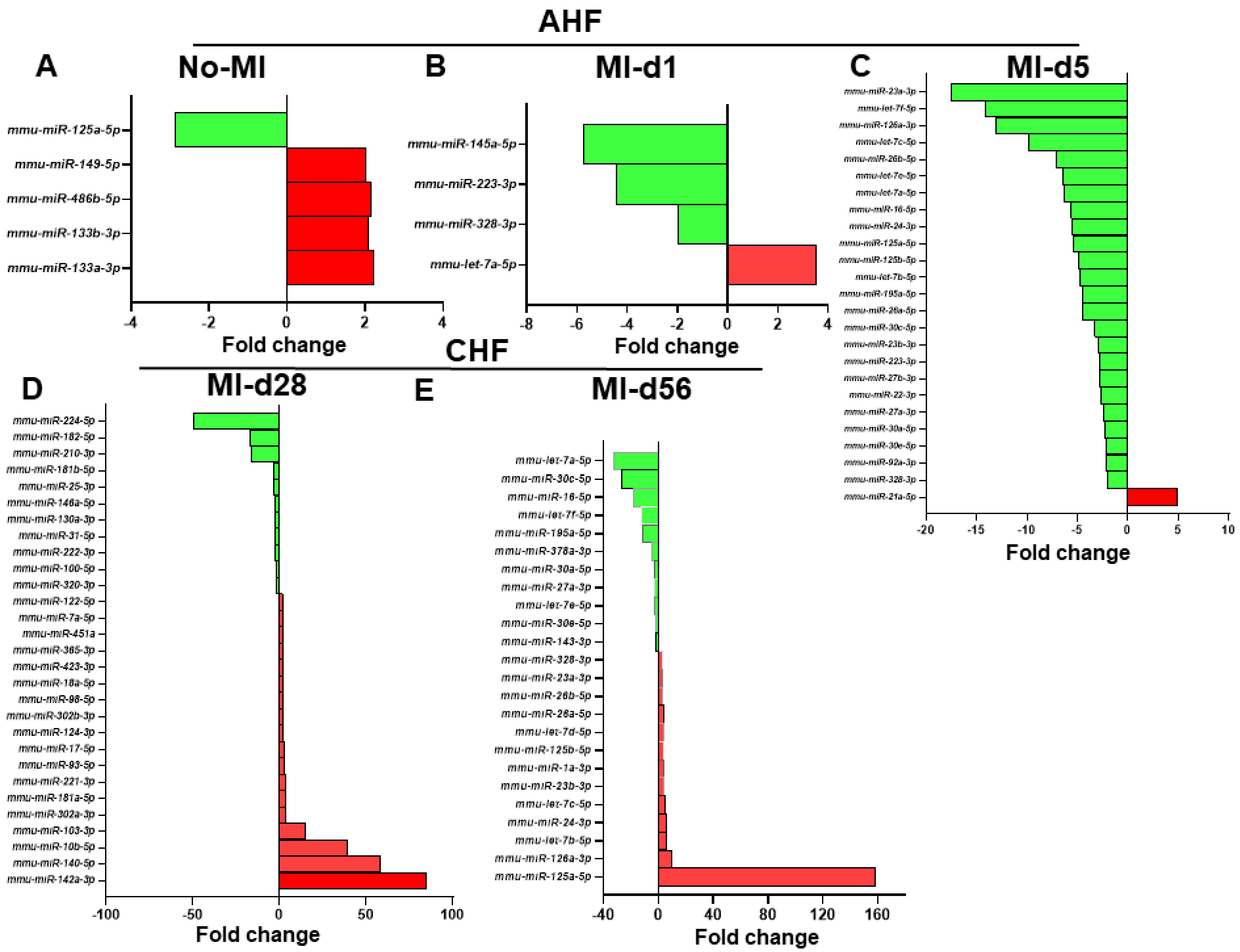
AHF
A
No-MI
mmu-miR-125a-5p
mmu-miR-149-5p
mmu-miR-486b-5p
mmu-miR-133b-3p
mmu-miR-133a-3p
Fold change
B
MI-d1
mmu-miR-145a-5p
mmu-miR-223-3p
mmu-miR-328-3p
mmu-let-7a-5p
Fold change
C
MI-d5
Fold change
CHF
D
MI-d28
Fold change
E
MI-d56
mmu-let-7a-5p
mmu-miR-30c-5p
mmu-miR-16-5p
mmu-let-7f-5p
mmu-miR-195a-5p
mmu-miR-378a-3p
mmu-miR-30a-5p
mmu-miR-27a-3p
mmu-let-7e-5p
mmu-miR-30e-5p
mmu-miR-143-3p
mmu-miR-328-3p
mmu-miR-23a-3p
mmu-miR-26b-5p
mmu-miR-26a-5p
mmu-let-7d-5p
mmu-miR-125b-5p
mmu-miR-1a-3p
mmu-miR-23b-3p
mmu-let-7c-5p
mmu-miR-24-3p
mmu-let-7b-5p
mmu-miR-126a-3p
mmu-miR-125a-5p
Fold change

2
